# Different gene rearrangements of the genus *Dardanus* (Anomura: Diogenidae) and insights into the phylogeny of Paguroidea

**DOI:** 10.1038/s41598-021-01338-8

**Published:** 2021-11-08

**Authors:** Ying Zhang, Lei Meng, Liming Wei, Xinting Lu, Bingjian Liu, Liqin Liu, Zhenming Lü, Yang Gao, Li Gong

**Affiliations:** 1 National Engineering Laboratory of Marine Germplasm Resources Exploration and Utilization, National Engineering Research Center for Facilitated Marine Aquaculture, Marine Science and Technology College, Zhejiang Ocean University, Zhoushan, 316022 China; 2 School of Fishery, Zhejiang Ocean University, Zhoushan, 316022 China

**Keywords:** Evolution, Molecular biology, Systems biology, Genetics

## Abstract

Complete mitochondrial genomes (mitogenomes) can provide useful information for phylogenetic relationships, gene rearrangement, and molecular evolution. In this study, the complete mitogenomes of two hermit crabs, *Dardanus arrosor* and *Dardanus aspersus*, were sequenced for the first time and compared with other published mitogenomes of Paguroidea. Each of the two mitogenomes contains an entire set of 37 genes and a putative control region, but they display different gene arrangements. The different arrangements of the two mitogenomes might be the result of transposition, reversal, and tandem duplication/random loss events from the ancestral pancrustacean pattern. Genome sequence similarity analysis reveals the gene rearrangement in 15 Paguroidea mitogenomes. After synteny analysis between the 15 Paguroidea mitogenomes, an obvious rearranged region is found in *D. aspersus* mitogenome. Across the 13 protein-coding genes (PCGs) tested, *COI* has the least and *ND6* has the largest genetic distances among the 15 hermit crabs, indicating varied evolution rates of PCGs. In addition, the dN/dS ratio analysis shows that all PCGs are evolving under purifying selection. The phylogenetic analyses based on both gene order and sequence data present the monophyly of three families (Paguridae, Coenobitidae, and Pylochelidae) and the paraphyly of the family Diogenidae. Meanwhile, the phylogenetic tree based on the nucleotide sequences of 13 PCGs shows that two *Dardanus* species formed a sister group with five Coenobitidae species. These findings help to better understand the gene rearrangement and phylogeny of Paguroidea, as well as provide new insights into the usefulness of mitochondrial gene order as a phylogenetic marker.

## Introduction

The metazoan mitochondrial genome (mitogenome) is typically a closed circular double-stranded DNA molecule. It is relatively small (15–20 kb) and contains 13 protein-coding genes (PCGs), 22 transfer RNA genes (tRNAs), two ribosomal RNA genes (12S and 16S), and an AT-rich region (also called control region, CR)^1^. Owing to some unique features like small genome size, conserved gene content, absence of introns, maternal inheritance, low level of recombination, and fast rate of evolution^2,3^, the mitogenome has been extensively used in population genetics, comparative genomics, and phylogenetic studies^4–6^. Besides, comparative analyses of the complete mitogenomes of closely related taxa can deepen the understanding of gene rearrangements and evolutionary relationships^7,8^.

Generally, the gene order in most vertebrate mitogenomes is considered conserved. However, extensive mitochondrial gene rearrangements have been observed in invertebrate mitogenomes, such as in cephalopods^9^, bivalves^10^, insects^11^, and crabs^12,13^. The gene rearrangement within a lineage has been supposed to be phylogenetically informative; therefore, comparative analysis of mitochondrial gene order has been proved to be a valuable phylogenetic tool. For example, Yuan et al.^14^ compared the mitochondrial gene arrangements of six heterodont bivalves and concluded that *Sanguinolaria* (Psammobiidae) is not monophyletic. By gene order comparisons of echinoderms, Smith et al.^15^ provided evidence that the sea cucumbers should group with sea urchins and sea stars with brittle stars. Besides, Morrison et al.^16^ reconstructed a phylogeny for decapod taxa based on gene rearrangements and DNA sequences. Their findings supported five independent origins of the crab-like form and confirmed the utility of mitochondrial gene rearrangements in phylogenetic reconstruction. So far, three main types of gene rearrangement events have been observed in the mitogenomes of animals, including translocation, shuffling, and inversion^17–20^. Correspondingly, four mechanisms have been proposed to account for mitogenomic rearrangements, including tandem duplication/random loss (TDRL) model^21^, tRNA mis-priming model^22^, intramitochondrial recombination model^23^, and tandem duplication/non-random loss model^24^.

The infraorder Anomura consists of approximately 2450 extant species^25^ with a broad range of ecological specializations and with various lineages having successfully adapted to freshwater, terrestrial, and diverse marine environments^6^. The latest classification scheme divides Anomura into Aegloidea, Chirostyloidea, Galatheoidea, Hippoidea, Lithodoidea, Lomisoidea, and Paguroidea. Hermit crabs (the superfamily Paguroidea Latreille, 1802) consist of Coenobitidae, Diogenidae, Paguridae, Parapaguridae, Pylochelidae and Pylojacquesidae, with more than 1100 species inhabiting diverse biotopes from intertidal to deep seas^26^. They represent an intermediate group of crustaceans from Macrura to Brachyura, which occupy an important position in crustacean evolution^27^. However, their evolutionary relationships at many taxonomic levels are still waiting for researchers to resolve^28–30^. Diogenidae Ortmann, 1892 is the second largest family of the superfamily Paguroidea. According to WoRMS (http://www.marinespecies.org/), the family Diogenidae has 22 genera and 482 species in total. Among them, the genus *Dardanus* Paulson, 1875 is one of the most diverse genera within Diogenidae. Previous studies of this genus mainly focused on morphology^31,32^, with almost no attention given to molecular phylogeny. To date, the phylogenetic location of the genus *Dardanus* has not been well resolved.

Accordingly, in the present study, we newly sequenced and annotated the complete mitogenomes of two hermit crabs, *D. arrosor* and *D. aspersus*. These are the first published mitogenomes of the genus *Dardanus*. Combined with 13 available Paguroidea mitogenomes, we performed a comparative mitogenomics analysis, in order to: (a) investigate the characteristics of Paguroidea mitogenomes; (b) estimate the phylogenetic information of mitochondrial gene rearrangements; (c) reconstruct the phylogeny of Paguroidea that could lay a foundation for further evolutionary studies.

## Materials and methods

### Sampling, DNA extraction, mitogenome sequencing, and assembly

Specimens of *D. arrosor* and *D. aspersus* were collected from Zhoushan Province, China (29° 45′ 32″ N, 121° 45′ 30″ E). Specimens were immediately preserved in 95% ethanol until DNA extraction. The SQ Tissue DNA Kit (OMEGA) was used to extract the total genomic DNA from muscle tissue following the manufacturer's instructions. The genomic DNA was sent to Shanghai Origingene Biopharm Technology Co., Ltd. for library preparation and high-throughput sequencing. The libraries were constructed by using the VAHTS Universal Plus DNA Library Prep Kit, with an insert size of 150 bp. Paired-end sequencing with a read length of 150 bp was performed on an Illumina Hiseq 6000 platform. Adapters and low-quality bases were removed using cutadapt v1.16^33^ with the following parameters: -q 20 -m 20. Trimmed reads shorter than 50 bp were discarded. Quality control of raw and trimmed reads was performed using FastQC v0.11.5 (http://www.bioinformatics.babraham.ac.uk/projects/fastqc/). The filtered clean data were assembled and mapped to complete mitogenome sequence using NOVOPlasty v2.7.2^34^.

### Mitogenome annotation and sequence analysis

The newly assembled mitogenomes of *D. arrosor* and *D. aspersus* were annotated using the software of Sequin (version 15.10, http://www.ncbi.nlm.nih.gov/Sequin/). The boundaries of protein-coding and ribosomal RNA genes were performed using NCBI-BLAST (http://blast.ncbi.nlm.nih.gov). Transfer RNA genes were manually plotted, according to the secondary structure predicted by the MITOS Web Server^35^ and tRNAscan-SE 1.21^36^. The control region was determined by the locations of adjacent genes. Finally, circular mitogenome maps of *D. arrosor* and *D. aspersus* were drawn with the BLAST Ring Image Generator (BRIG) v0.95^37^.

The base composition and relative synonymous codon usage (RSCU) were obtained using MEGA X^38^. The strand asymmetry was calculated using the following formulas: AT-skew = (A − T)/(A + T); GC-skew = (G − C)/(G + C)^39^. Furthermore, we chose the complete mitogenome of *Pagurus filholi* as the reference genome for comparative genomic analysis. Genome sequence similarity among the 15 Paguroidea species was carried out using the BRIG tool. Synteny analysis between the genomes was performed using Mauve v2.4.0^40^. To estimate the evolutionary-selection constraints on 13 PCGs in the superfamily Paguroidea, the nonsynonymous (dN) and synonymous (dS) substitution rates were calculated using Mega X. The genetic distances of 13 PCGs were also estimated using Mega X based on the Kimura 2-parameter (K2P) substitution model.

### Mitochondrial gene order comparisons and phylogeny

CREx^41^ was used to compare the mitochondrial gene order and infer the gene rearrangement scenarios based on common intervals. CREx considers four types of rearrangement events: reversals (R), transpositions (T), reverse transpositions (RT), and tandem-duplication-random-losses (TDRL). MLGO web server^42^ was used to infer a phylogeny from gene order data.

### Phylogenetic analysis

Phylogeny of the Paguroidea was inferred based on 13 available complete mitogenomes expanded with the two newly determined ones (Table 1). The species *Helicana wuana* and *H. latimera* from Grapsoidea were used as outgroups. Fasta files with the nucleotide sequences for all 13 PCGs were extracted from the GenBank files using PhyloSuite^43^. The MAFFT program^44^ integrated into PhyloSuite was executed to align multiple sequences in normal-alignment mode, and ambiguously aligned regions were identified and moved by Gblocks^45^. Subsequently, the sequences were concatenated into a single alignment and converted into input files (Phylip and Nexus format) for phylogenetic analyses. Phylogenetic trees were built under maximum likelihood (ML) and Bayesian inference (BI) methods. The ML analysis was conducted using IQ-TREE^46^, under an ML + rapid bootstrap (BS) algorithm with 1000 replicates. The BI analysis was conducted in MrBayes 3.2.6^47^ with 3 × 10^6^ metropolis-coupled Markov Chain Monte Carlo (MCMCMC) generations, sampling every 1000 generations, and the first 25% of the generations were discarded as burn-in. To guarantee the stationarity had been reached, the average standard deviation of split frequencies was set below 0.01.

**Table 1:** Basic information of 15 Paguroidea species and two outgroups used in this paper.

Species	Family	Superfamily	Length (bp)	Accession No	References
*Pagurus filholi*	Paguridae	Paguroidea	15,674	LC222528	^48^
*Pagurus japonicus*	Paguridae	Paguroidea	16,401	LC222532	^48^
*Pagurus gracilipes*	Paguridae	Paguroidea	16,051	LC222534	^48^
*Pagurus maculosus*	Paguridae	Paguroidea	15,420	LC222524	^48^
*Pagurus nigrofascia*	Paguridae	Paguroidea	15,423	LC222531	^48^
*Pagurus longicarpus*	Paguridae	Paguroidea	15,630	NC_003058	^49^
*Coenobita rugosus*	Coenobitidae	Paguroidea	16,427	KY352235	^6^
*Coenobita variabilis*	Coenobitidae	Paguroidea	16,421	KY352236	^6^
*Coenobita perlatus*	Coenobitidae	Paguroidea	16,447	KY352234	^6^
*Coenobita brevimanus*	Coenobitidae	Paguroidea	16,393	NC_050386	^50^
*Birgus latro*	Coenobitidae	Paguroidea	16,411	NC_045091	^51^
*Dardanus arrosor*	Diogenidae	Paguroidea	16,592	MW147148	This study
*Dardanus aspersus*	Diogenidae	Paguroidea	16,916	MW715812	This study
*Clibanarius infraspinatus*	Diogenidae	Paguroidea	16,504	NC_025776	^52^
*Pylocheles mortensenii*	Pylochelidae	Paguroidea	15,093	KY352242	^6^
*Helice wuana*	Varunidae	Grapsoidea	16,359	NC_034995	Outgroup
*Helice latimera*	Varunidae	Grapsoidea	16,246	NC_033865	Outgroup

## Results

### General features of *D. arrosor* and *D. aspersus* mitogenomes

The complete mitogenomes of *D. arrosor* and *D. aspersus* are 16,592 bp and 16,916 bp in length, respectively (GenBank accessions MW147148 and MW715812) (Figs. S1, S2, Tables 1, 2). Each mitogenome contains a typical set of 37 genes (13 PCGs, 22 tRNAs, and two rRNAs) and a putative CR. Within these genes, eight PCGs (*COI*, *COII*, *ND2*, *ATP8*, *ATP6*, *COIII*, *ND6*, and *Cyt b*) and 11 tRNAs (*tRNA-Leu*^TAG^, *Lys*, *Met*, *Ile*, *Asp*, *Arg*, *Asn*, *Glu*, *Thr*, *Ser*^TGA^, and *Tyr*) are encoded by the heavy (H-) strand, while five PCGs (*ND5*, *ND4*, *ND4L*, *ND1*, and *ND3*), 11 tRNAs (*tRNA-Phe*, *His*, *Pro*, *Val*, *Ser*^TCT^, *Ala*, *Gly*, *Leu*^TAA^, *Trp*, *Gln*, and *Cys*), and two rRNAs (*16S* and *12S rRNA*) are encoded by the light (L-) strand. But the gene order of the two mitogenomes is different.

**Table 2:** Features of the mitochondrial genome of *D. arrosors.*

Gene	Position		Length (bp)	Amino acid	Start/stop codon	Anticodon	Intergenic region	Strand
	From	To						
*COI*	1	1539	1539	512	ATG/TAA		− 5	H
*Leu* (*L*_1_)	1535	1597	63			TAG	4	H
*COII*	1602	2294	693	230	ATG/TAG		6	H
*Lys* (*K*)	2301	2367	67			TTT	8	H
*Met* (*M*)	2376	2443	68			CAT	7	H
*Ile* (*I*)	2451	2514	64			GAT	52	H
*ND2*	2567	3568	1002	333	ATT/TAG		4	H
*Asp* (*D*)	3573	3637	65			GTC	0	H
*ATP8*	3638	3796	159	52	ATT/TAG		− 7	H
*ATP6*	3790	4464	675	224	ATG/TAA		− 1	H
*COIII*	4464	5255	792	263	ATG/TAA		11	H
*Arg* (*R*)	5267	5333	67			TCG	− 1	H
*Asn* (*N*)	5333	5397	65			GTT	3	H
*Glu* (*E*)	5401	5466	66			TTC	1	H
*Phe* (*F*)	5468	5532	65			GAA	8	L
*ND5*	5541	7260	1720	573	ATG/T		0	L
*His* (*H*)	7261	7325	65			GTG	64	L
*ND4*	7390	8820	1431	476	ATG/TAA		− 7	L
*ND4L*	8814	9116	303	100	ATG/TAA		2	L
*Thr* (*T*)	9119	9185	67			TGT	12	H
*ND6*	9198	9716	519	172	ATG/GAC		− 5	H
*Cyt b*	9712	10,848	1137	378	ATG/TAG		− 1	H
*Ser* (*S*_2_)	10,848	10,913	66			TGA	2	H
*Pro (P*)	10,916	10,982	67			TGG	3	L
*ND1*	10,986	11,915	930	309	ATT/TAG		0	L
*16S*	11,916	13,322	1407				0	L
*Val* (*V*)	13,323	13,391	69			TAC	1	L
*12S*	13,393	14,190	798				0	L
CR	14,191	15,686	1496				0	H
*Ser* (*S*_1_)	15,687	15,753	67			TCT	0	L
*Ala* (*A*)	15,754	15,815	62			TGC	5	L
*ND3*	15,821	16,168	348	115	ATT/TAG		3	L
*Gly* (*G*)	16,172	16,237	66			TTC	1	L
*Leu* (*L*_2_)	16,239	16,304	66			TAA	0	L
*Tyr* (*Y*)	16,305	16,373	69			GTA	8	H
*Trp* (*W*)	16,382	16,450	69			TCA	0	L
*Gln* (*Q*)	16,451	16,515	65			TTG	8	L
*Cys* (*C*)	16,524	16,590	67			GCA	1	L

There are 214 intergenic nucleotides (IGNs) dispersed in 22 locations for *D. arrosor*, 596 IGNs in 22 locations for *D. aspersus*. The longest IGN is 64 bp (between *tRNA-His* and *ND4*) and 176 bp (between *ND4L* and *tRNA-Pro*) for *D. arrosor* and *D. aspersus*, respectively (Tables 2, 3). Meanwhile, 27 overlapping nucleotides are located in seven pairs of neighboring genes for both mitogenomes. These overlapping nucleotides vary in length from 1 to 7 bp, and the longest overlap is located between *ATP8* and *ATP6* as well as *ND4* and *ND4L* (Tables 2, 3). The base composition of *D. arrosor* is A = 33.3%, T = 34.6%, C = 15.7%, G = 16.4% and that of *D. aspersus* is A = 33.4%, T = 32.6%, C = 15.7%, G = 18.3%. The AT content is 67.9% in *D. arrosor* and 66.0% in *D. aspersus*, thus exhibiting a strong AT bias (Tables S1, S2).

**Table 3:** Features of the mitochondrial genome of *D. asperses.*

Gene	Position		Length (bp)	Amino acid	Start/stop codon	Anticodon	Intergenic region	Strand
	From	To						
*COI*	1	1539	1539	512	ATG/TAA		− 5	H
*Leu* (*L*_1_)	1535	1599	65			TAG	5	H
*COII*	1605	2297	693	230	ATG/TAG		8	H
*Lys* (*K*)	2306	2373	68			TTT	9	H
*Met* (*M*)	2383	2449	67			CAT	7	H
*Ile* (*I*)	2457	2523	67			GAT	51	H
*ND2*	2575	3576	1002	333	ATT/TAA		3	H
*Asp* (*D*)	3580	3645	66			GTC	0	H
*ATP8*	3646	3804	159	52	ATC/TAG		− 7	H
*ATP6*	3798	4472	675	224	ATG/TAA		− 1	H
*COIII*	4472	5263	792	263	ATG/TAG		17	H
*Arg* (*R*)	5281	5346	66			TCG	0	H
*Asn* (*N*)	5347	5408	62			GTT	6	H
*Glu* (*E*)	5415	5480	66			TTC	163	H
*Thr* (*T*)	5644	5711	68				9	H
*ND6*	5721	6239	519		ATG/GAC		− 5	H
*Cyt b*	6235	7371	1137		ATG/TAG		− 1	H
*Ser* (*S*_2_)	7371	7438	68				3	H
*Phe* (*F*)	7442	7504	63			GAA	3	L
*ND5*	7508	9233	1726	575	GTG/T		0	L
*His* (*H*)	9234	9299	66			GTG	87	L
*ND4*	9387	10,790	1345	467	ATG/TAA		− 7	L
*ND4L*	10,784	11,086	303	100	ATG/TAA		176	L
*Pro (P*)	11,263	11,329	67			TGG	2	L
*ND1*	11,332	12,261	930	309	ATC/TAA		− 1	L
*16S*	12,261	13,660	1400				0	L
*Val* (*V*)	13,661	13,729	69			TAC	0	L
*12S*	13,730	14,530	801				0	L
CR	14,531	15,991	1461				0	H
*Ser* (*S*_1_)	15,992	16,054	63			TCT	6	L
*Ala* (*A*)	16,061	16,125	65			TGC	8	L
*ND3*	16,134	16,478	345	114	TTG/TAG		6	L
*Gly* (*G*)	16,485	16,550	66			TTC	9	L
*Leu* (*L*_2_)	16,560	16,627	68			TAA	0	L
*Tyr* (*Y*)	16,628	16,697	70			GTA	8	H
*Trp* (*W*)	16,706	16,775	70			TCA	0	L
*Gln* (*Q*)	16,776	16,838	63			TTG	9	L
*Cys* (*C*)	16,848	16,914	67			GCA	1	L

Except for *ND5* (uses GTG as the start codon) and *ND3* (uses TTG as the start codon) in *D. aspersus* mitogenome (Tables 2, 3), the remaining PCGs initiate with typical ATN codons. As for the stop codon, the majority of PCGs stop with TAA or TAG except for *ND5* (uses a single T as the stop codon) and *ND6* (uses GAC as the stop codon) in the two mitogenomes (Tables 2, 3). The GC-skew values of five PCGs (*ND5*, *ND4*, *ND4L*, *ND1*, and *ND3*) are positive, indicating they are encoded by the L-strand, whereas the remaining eight exhibit negative values, indicating they are encoded by the H-strand (Tables S1, S2).

Twenty-two tRNAs of *D. arrosor* and *D. aspersus* mitogenomes are scattered throughout the entire mitogenome (Tables 2, 3). The total length of 22 tRNAs is 1455 bp in *D. arrosor* and 1460 bp in *D. aspersus* (Tables S1, S2). All of the tRNAs can be folded into typical cloverleaf secondary structures except for the *tRNA-Ser* (TCT) in the two mitogenomes (Figs. S3, S4). The lack of DHU arm in *tRNA-Ser* (TCT) is thought to be a common phenomenon in metazoan mitogenomes^12,53^. The *16S rRNA* and *12S rRNA* genes of *D. arrosor* and *D. aspersus* are located between *ND1* and *tRNA-Val* and between *tRNA-Val* and CR, respectively. The AT content of the two rRNAs is 73.3% in *D. arrosor*, which is higher than that of *D. aspersus* (70.1%) (Tables S1, S2).

### Codon usage bias in Paguroidea mitogenomes

Codon usage bias is a phenomenon in which specific codons are used more frequently than other synonymous codons by certain organisms during the translation of genes to proteins. In this study, the relative synonymous codon usage (RSCU) of 15 hermit crabs is roughly identical. Except for *Pagurus longicarpus* and *Pylocheles mortensenii*, which miss codons, the other 13 species have all 62 available codons. The lost codons usually belong to GC-rich codon-families (Fig. S5, Table S3). The RSCU values for the codons NNU and NNA are usually greater than one, suggesting a strong AT bias in the third codon position (Fig. S5, Table S3). This result supports the hypothesis that the codon usage bias in PCGs and the AT bias of the third codon position are positively correlated^54,55^.

### Comparative genomic analysis of Paguroidea species

**Figure 1:**
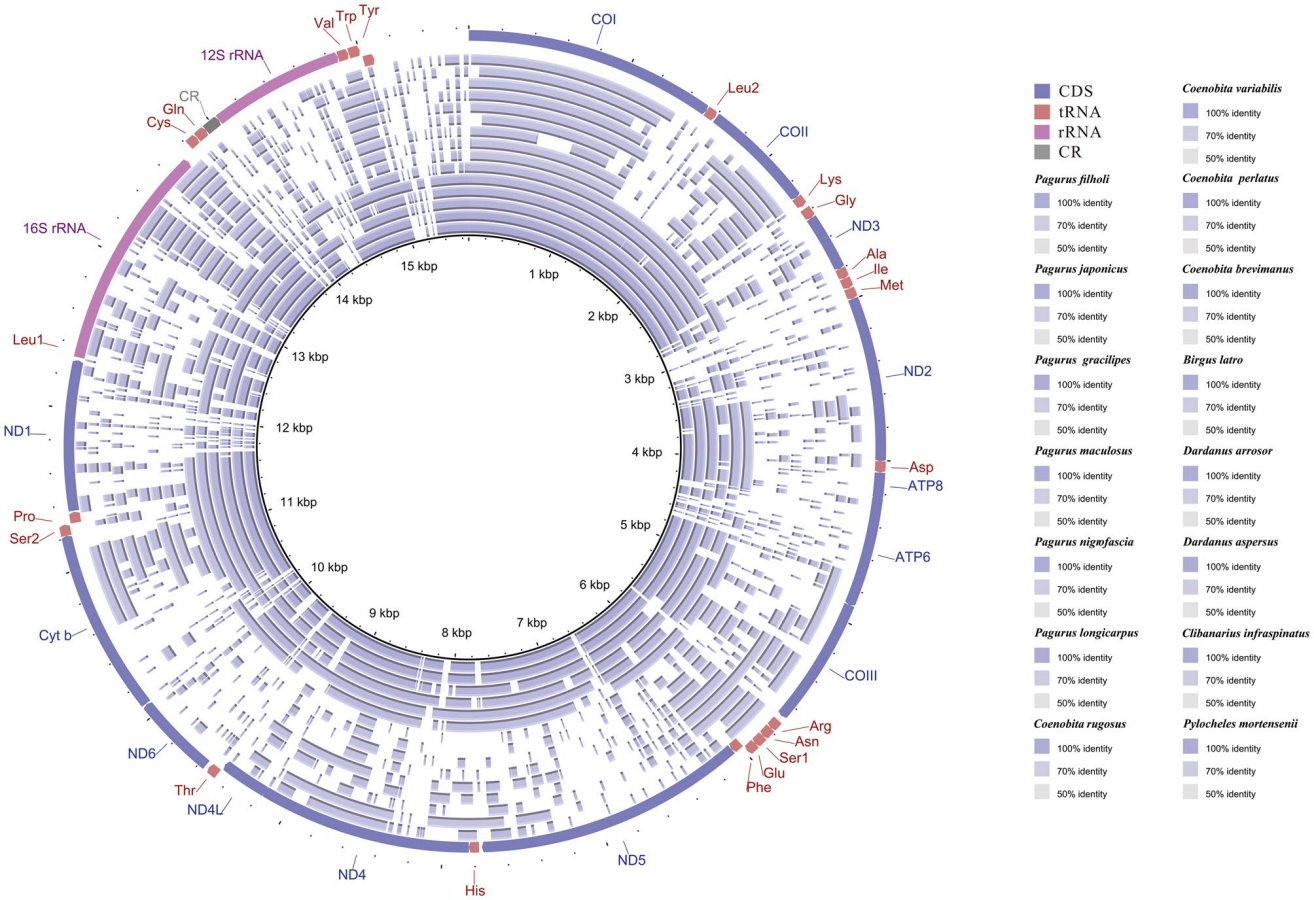
BLAST comparison of all Paguroidea mitogenomes against a reference (*P. filholi*) generated by Blast Ring Image Generator (BRIG). The intensity of the ring color denotes the degree of sequence conservation at that region. Gaps in rings correspond to regions with less than 50% identity to the reference sequence. The innermost ring to the outermost in this figure is presented as follows: *P. filholi*, *P. japonicus*, *P. gracilipes*, *P. maculosus*, *P. nigrofascia*, *P. longicarpus*, *Coenobita rugosus*, *C. variabilis*, *C. perlatus*, *C. brevimanus*, *Birgus latro*, *D. arrosor*, D. aspersus, *Clibanarius infraspinatus*, and *P. mortensenii*.

Using the *P. filholi* mitogenome as the reference sequence, all available mitogenomes in the superfamily Paguroidea were compared using BRIG. The results reveal the gene rearrangement in 15 Paguroidea mitogenomes (Fig. 1). The mitogenomes of the family Paguridae are observed to be fairly conserved, with about 80% sequence identity in most regions (six innermost rings in Fig. 1). However, the mitogenomes of the species under the families Coenobitidae, Diogenidae, and Pylochelidae are quite different from the family Paguridae, as can be seen from the larger gap regions in the BRIG map (nine outermost rings in Fig. 1).

**Figure 2:**
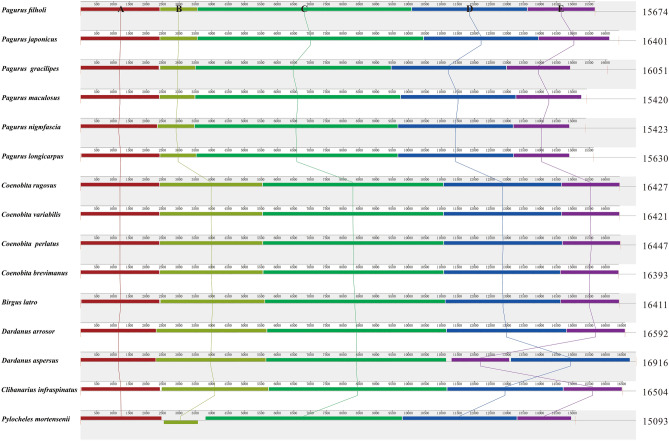
Multiple genome alignments of 15 hermit crabs. The mitogenome of *P. filholi* is shown at the top as the reference genome. All genomes are started from the *Pro* gene. The ruler at the top of each genome shows nucleotide positions. Within each of the alignments, local collinear blocks are represented by blocks of the same color connected by lines.

By using the Mauve analysis, we identified five large genomic homologous regions (marked A–E in Fig. 2). These homologous regions are commonly presented in all 15 Paguroidea mitogenomes. For the B region, it has the maximum length diversification and is greatly contributed to the genome size variation between Paguroidea mitogenomes (Fig. 2). In addition, we found that homologous regions D and E are rearranged in *D. aspersus* mitogenome. The two homologous regions show an E–D order in *D. aspersus* mitogenome, while the other hermit crabs display a D–E order (Fig. 2).

**Figure 3:**
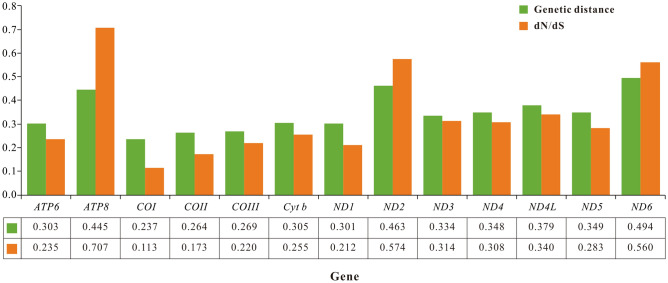
Genetic distance (on average) and dN/dS substitution rates of 13 PCGs among 15 hermit crabs.

To estimate the evolutionary-selection constraints on 13 PCGs in the superfamily Paguroidea, we perform dN/dS analysis for each PCG. The dN/dS ratios for all PCGs are less than 1, indicating that these genes are evolving primarily under purifying selection. Among them, the lowest dN/dS value (0.113) for *COI* gene indicates the strongest purifying selection, whereas the highest dN/dS value (0.707) for *ATP8* gene shows a highly relaxed purifying selection (Fig. 3). In general, the dN/dS values indicate that the evolution of Paguroidea mitogenomes has been dominated by purifying selection. Besides, we conduct genetic distance analysis for 13 PCGs. *COI* gene possesses the least genetic distance (average 0.237), and *ND6* gene captures the largest value (average 0.494), representing the most conserved and variable genes, respectively (Fig. 3).

### Mitochondrial gene order and rearrangements

**Figure 4:**
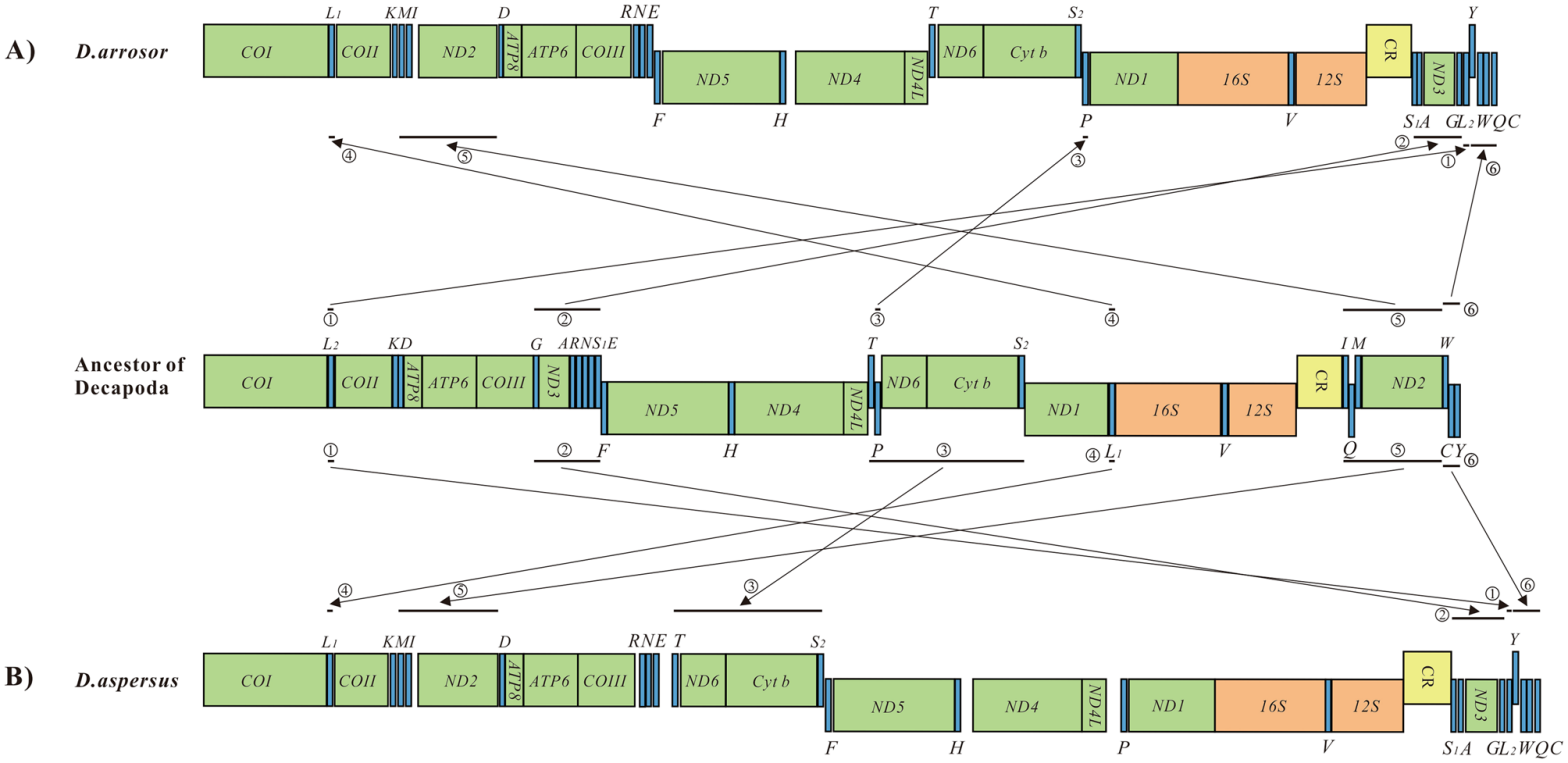
(**A**) Gene rearrangements in *D. arrosor* mitogenome; (**B**) Gene rearrangements in *D. aspersus* mitogenome.

The gene arrangement in the mitogenomes of *D. arrosor* and *D. aspersus* is shown in Fig. 4. The gene order of the two mitogenomes belonging to the same genus is different. Compared with the gene order in ancestral crustaceans (the pancrutacean ground pattern) mitogenomes^56^, the gene order in *D. arrosor* and *D. aspersus* mitogenomes underwent large-scale gene rearrangements. For *D. arrosor*, at least six gene clusters (or genes) significantly differ from the typical order, involving 12 tRNAs (*L*_2_, *G*, *A*, *S*_1_, *P*, *L*_1_, *I*, *Q*, *M*, *W*, *C*, and *Y*), and two PCGs (*ND3* and *ND2*). Of these six gene rearrangements, a single *L*_2_ is inverted from the downstream of *COI* in the H-strand to downstream of the *G* in the L-strand (Fig. 4A①). The *G*- *ND3*- *A*- *S*_1_ cluster is inverted from the downstream of *COIII* in the H-strand to downstream of the CR in the L-strand (Fig. 4A②). A single *P* moves from the downstream of *T* to downstream of the *S*_2_ (Fig. 4A③). A single *L*_1_ is inverted from the downstream of *ND1* in the L-strand to downstream of the *COI* in the H-strand (Fig. 4A④). The *I*- *Q*- *M*- *ND2* cluster is divided into two sections, one (*I*, *M*, and *ND2*) is shifted to downstream of *K*. The other (*Q*) is shifted to the end of the linear mitogenome (Fig. 4A⑤). The *W*- *C*- *Y* cluster order is changed into *Y*- *W*- *C* order (Fig. 4A⑥). For *D. aspersus*, there are also at least six gene clusters (or genes) that differ significantly from the typical order, but the genes involved are different from *D. arrosor*. The rearrangement process involves 14 tRNAs (*L*_2_, *G*, *A*, *S*_1_, *T*, *P*, *S*_2_, *L*_1_, *I*, *Q*, *M*, *W*, *C*, and *Y*), and four PCGs (*ND3*, *ND6*, *Cyt b*, and *ND2*). Relative to the gene arrangement of *D. arrosor* mitogenome, the *T*- *P*- *ND6*- *Cyt b*- *S*_2_ cluster is divided into two sections, one (*T*, *ND6*, *Cyt b*, and *S*_2_) is shifted to downstream of *E*. The other (*P*) is shifted to downstream of *ND4L* (Fig. 4A③). Based on the CREx analysis, transposition, reversal, and TDRL may be involved in the large-scale gene rearrangements in *D. arrosor* and *D. aspersus* mitogenomes (Figs. S6, S7).

**Figure 5:**
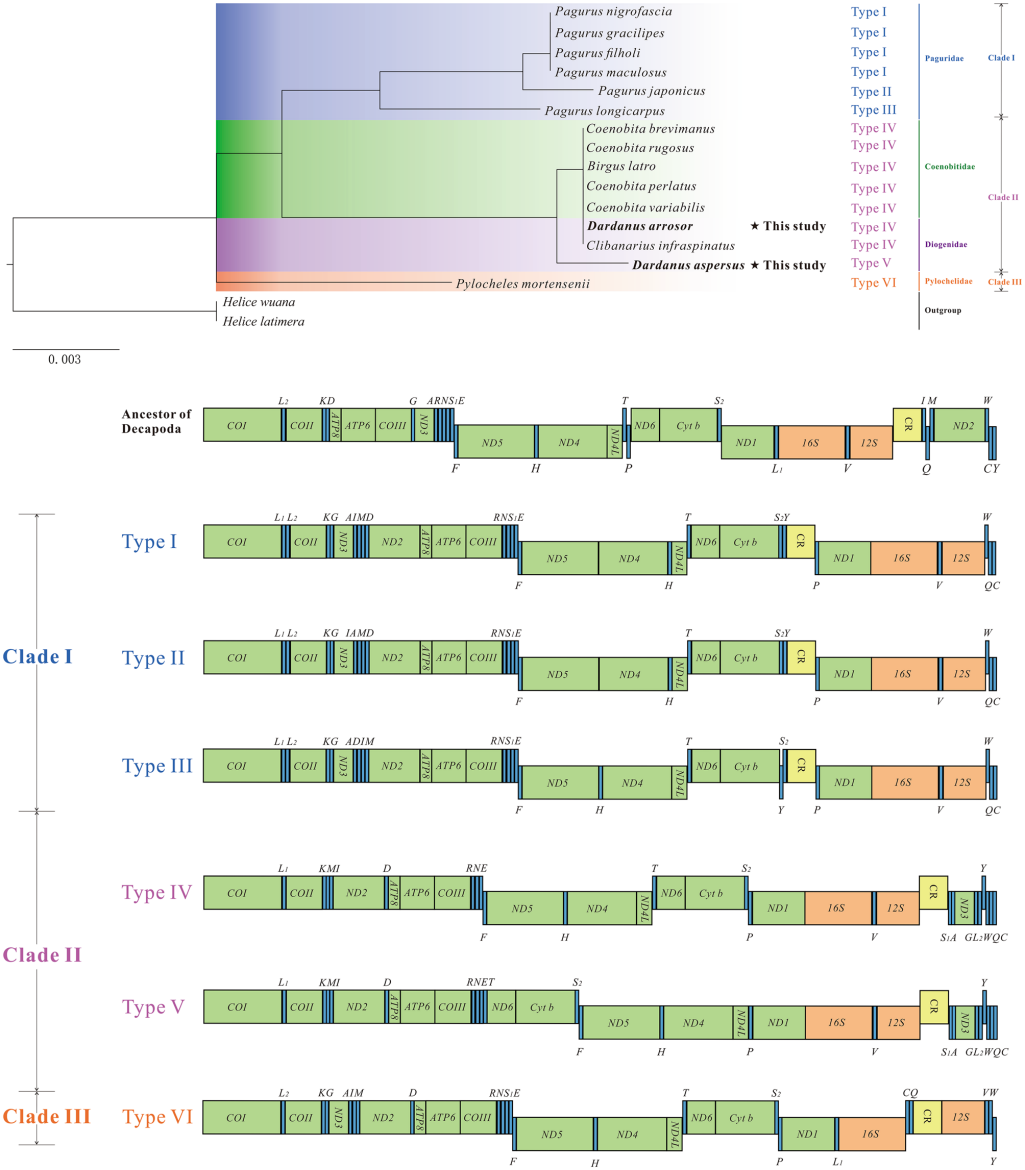
Phylogeny of Paguroidea reconstructed using gene order data, and arrangement of mitochondrial genes in the ancestral pancrustacean pattern and the superfamily Paguroidea. PCGs and CR are indicated with boxes, and tRNAs are indicated with columns. *COI* has been designated the start point for the linear representation of the gene arrangement.

The 15 hermit crabs exhibit six types of gene organization (Fig. 5). The mitogenomes of the family Paguridae possess three types of gene order (Type I, Type II, and Type III in Fig. 5). Relative to the remaining three types of gene order (Type IV, Type V, and Type VI in Fig. 5), these three types of gene order are more similar. The mitogenomes of the family Diogenidae possess two types of gene order (Type IV and Type V in Fig. 5). Only the gene arrangement of one gene cluster (*T*- *ND6*- *Cyt b*- *S*_2_) is found to be different between the two gene orders. For the remaining two families, Coenobitidae and Pylochelidae, each has only one type of gene order (Type IV and Type VI in Fig. 5). Among them, Coenobitidae shares one of the two gene orders of Diogenidae (Type IV). These results are consistent with the conclusion from the gene order-based phylogenetic tree (Fig. 5). In the gene order tree, all Paguridae species cluster into a clade, showing the closest relationship (Clade I). Species of the family Coenobitidae and Diogenidae are clustered together as a group (Clade II). As the only representative of the family Pylochelidae (Clade III), *P. mortensenii* forms a seperate branch. Our results support that comparisons of mitochondrial gene rearrangements, to some extent, are a useful tool for phylogenetic studies.

### Phylogenetic analysis

**Figure 6:**
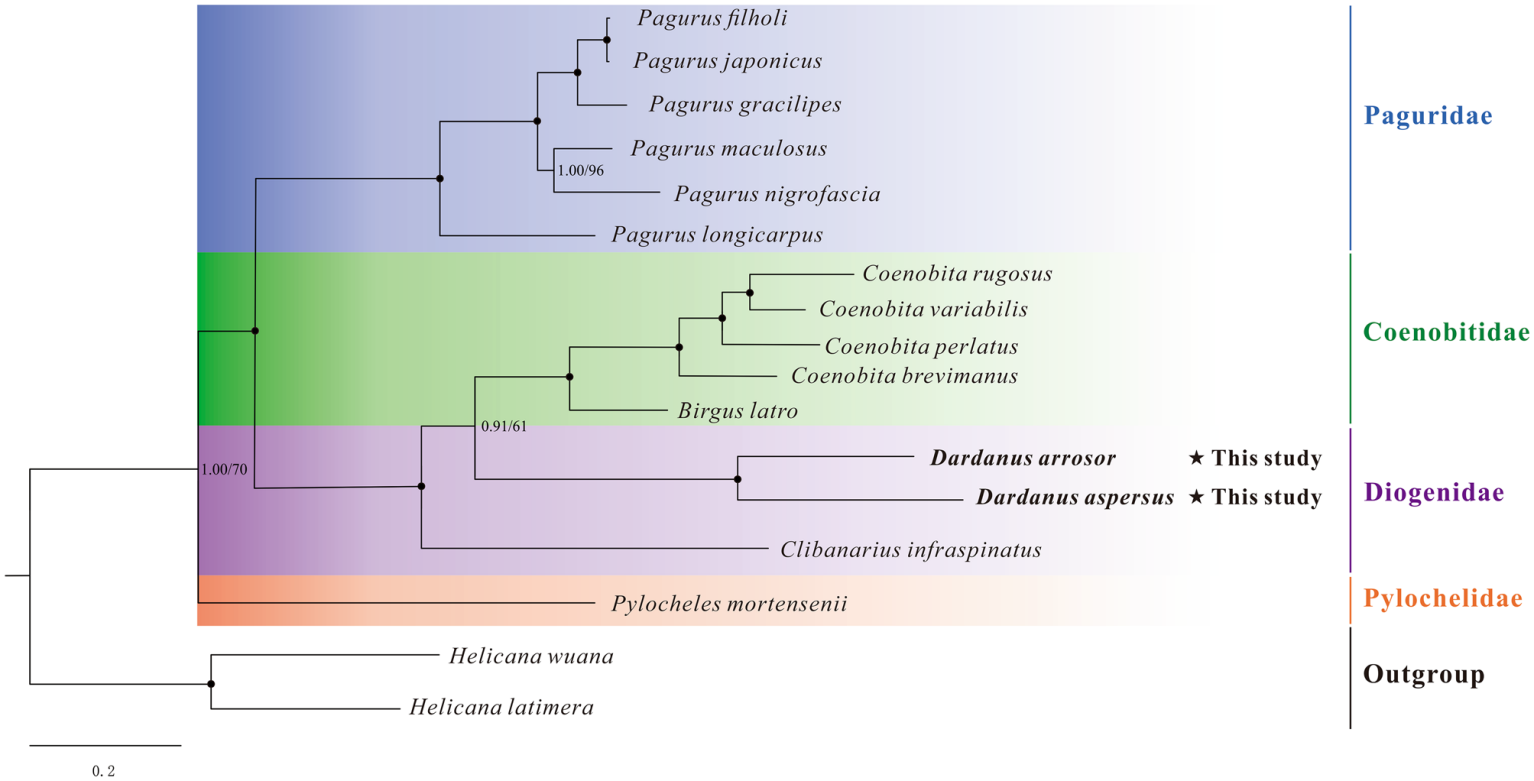
Phylogenetic tree of Paguroidea species inferred from the nucleotide sequences of 13 PCGs based on Bayesian inference (BI) and maximum likelihood (ML) analysis. Node marked with a solid circle indicates 100 maximum likelihood bootstrap value and 100% supporting value.

In the present study, the phylogenetic relationships among Paguroidea were reconstructed based on the nucleotide sequences of 13 PCGs using maximum likelihood (ML) and Bayesian (BI) methods. The phylogenetic trees (ML tree and BI tree) show an identical topology; thus, only one topology (BI) with both support values is displayed (Fig. 6). The results show that *D. arrosor* and *D. aspersus* are most closely related, forming part of the family Diogenidae. Three Diogenidae species are separated into two clades, two *Dardanus* species cluster together as a clade, and then form a sister group with five Coenobitidae species. While the remaining one (*Clibanarius infraspinatus*) forms a separate clade, resulting in the paraphyly of Diogenidae. Besides, of the four families included in the phylogenetic tree, almost all families except Diogenidae form a monophyletic clade. However, the paraphyly of the family Pylochelidae was originally proposed by Richter and Scholtz^57^ and has been confirmed by many previous researches^58,59^. Since there is only one representative of the family Pylochelidae in our study, the monophyly of this taxon should be treated with caution.

## Discussion

In the present study, our phylogenetic reconstruction based on the nucleotide sequences of 13 PCGs recovered a robust tree (Fig. 6). For a long time, the phylogenetic status of Diogenidae has been controversial. Most previous researches based on morphological features considered it to be a paraphyletic clade. But Forest^60,61^ suggested that the family Diogenidae is an ancient monophyletic group. In recent years, an increasing number of molecular studies, including ours, have supported the paraphyly of this taxon. For example, Tsang et al.’s used two nuclear protein genes to conduct phylogenetic inference and clearly pointed out that the family Diogenidae is a paraphyletic clade^62^. In their study, the genus *Coenobita* (Coenobitidae) is embedded within the branch of the family Diogenidae. Based on mitochondrial gene sequences, Landschoff and Gouws’s research recovered the paraphyly of the family Diogenidae as well^63^. However, there are few studies on the phylogenetic relationships among the genera of the family Diogenidae. Previous studies mainly focused on exploring the phylogenetic relationships of the infraorder Anomura, involving only a few genera and species of the family Diogenidae. Accordingly, increased taxon sampling is required to conclusively resolve the phylogenetic relationships within the family Diogenidae and the superfamily Paguroidea.

Besides, we reconstructed the phylogeny of Paguroidea based on the gene order (Fig. 5). In the family-level relationships within Paguroidea, the gene order information seems to be reliable for phylogenetic inference. A good illustration is that the monophyly of three families (Paguridae, Coenobitidae, and Pylochelidae) and the paraphyly of the family Diogenidae are reconfirmed in the gene order tree (Figs. 5, 6). Our conclusion is in accordance with previous workers, who suggested that gene rearrangements, to some extent, contain phylogenetic information. For example, Shao et al.^64^ compared the mitochondrial gene arrangements of 12 anomurans and found that *Munidopsis lauensis* and *M. verrilli* are most closely related to *Shinkaia crosnieri*. Based on the comparative analysis of mitochondrial gene arrangement within Coleoidea, Akasaki et al.^65^ concluded that order Octopoda might be the most ancestral among this subclass Coleoidea. However, the potential to resolve the phylogenetic relationships within families based on gene order alone is clearly inferior to sequence-based approaches. One example is that the monophyly of two genera (*Dardanus* and *Coenobita*) is not recovered in the gene order tree (Fig. 5). In future studies, it may be possible to resolve some long-standing phylogenetic controversies by integrating gene order and sequence data.

For most families of the order Decapoda, congeners belonging to the same family share the same gene arrangement generally. Accordingly, it is acceptable to apply gene rearrangement as a molecular marker for phylogenetic inference^6,15,16^. However, there are some exceptions. For example, the family Camptandriidae Stimpson, 1858 possess two different gene arrangements (unpublished), and the freshwater crabs Potamidae Ortmann, 1896 possess at least nine main types of gene rearrangement^66^. In the present study, we even found that two closely related species of the genus *Dardanus* capture different gene rearrangements. These examples challenge the utility of gene rearrangement as a molecular marker in phylogenetic studies. So it triggers a thought-provoking question that why the mitogenome gene arrangement differs between very closely related species? One possible hypothesis is that the mitogenome gene rearrangement is a continuous and dynamic process and may occur very recently even after speciation events. In future studies, more relevant data are essential to verify this hypothesis.

## Conclusion

In the present study, we reported the mitogenomes of *D. arrosor* and *D. aspersus*, supplementing the limited mitogenome information of the family Diogenidae (Anomura: Paguroidea). By analyzing the mitogenomes of *D. arrosor* and *D. aspersus* and comparing them with other published Paguroidea mitogenomes, we can draw the following conclusions: (a) the gene content of the two mitogenomes belonging to the same genus is conserved, whereas the gene arrangement is different; (b) CREx analysis reveals that transposition, reversal, and TDRL may be involved in the large-scale gene rearrangements in *D. arrosor* and *D. aspersus* mitogenomes; (c) the dN/dS analysis indicates that the evolution of Paguroidea mitogenomes has been dominated by purifying selection; (d) the phylogenetic analyses based on both gene order and sequence data reveal the monophyly of three families (Paguridae, Coenobitidae, and Pylochelidae) and the paraphyly of the family Diogenidae. In future studies, large-scale taxonomic samplings are still needed to further investigate the taxonomical and phylogenetic studies of Paguroidea.

## Supplementary Information

Supplementary Information.
