# Supplementary material for: Different gene rearrangements of the genus Dardanus (Anomura: Diogenidae) and insights into the phylogeny of Paguroidea
Source: Sci Rep. 2021 Nov 8;11:21833. doi: 10.1038/s41598-021-01338-8 (PMC8576005; doi:10.1038/s41598-021-01338-8)
Supplement: Supplementary file 1 — Supplementary Information. [file 41598_2021_1338_MOESM1_ESM.pdf]

Different gene arrangements of the genus *Dardanus* (Anomura: Paguroidea: Diogenidae) and insights into the phylogeny of Paguroidea

Ying Zhang[1], Lei Meng[2], Liming Wei[1], Xinting Lu[1], Bingjian Liu[1], Liqin Liu[1], Zhenming Lü[1], Yang Gao[2], Li Gong[1*]

1. National Engineering Laboratory of Marine Germplasm Resources Exploration and Utilization, National Engineering Research Center for Facilitated Marine Aquaculture, Marine Science and Technology College, Zhejiang Ocean University, Zhoushan 316022, China;
2. School of Fishery, Zhejiang Ocean University, Zhoushan 316022, China

[*] Corresponding author. E-mail address: gongli1027@163.com; gongli@zjou.edu.cn (Li Gong)

Table S1. Composition and skewness of *D. arrosor* mitogenome.

	A%	T%	G%	C%	(A + T) %	AT-skew	GC-skew	Length(bp)
Mitogenome	33.3	34.6	15.7	16.4	67.9	−0.018	−0.023	16,592
PCGs	27.0	40.3	16.5	16.3	67.2	−0.197	0.007	11248
COI	26.3	38.0	18.8	17.0	64.3	−0.183	0.051	1539
COII	28.0	37.5	18.5	16.0	65.5	−0.145	0.071	693
ND2	26.1	44.6	14.0	15.3	70.8	−0.261	−0.044	1002
ATP8	37.1	39.0	10.1	13.8	76.1	−0.025	−0.158	159
ATP6	27.0	38.4	15.4	19.3	65.3	−0.175	−0.111	675
COIII	25.5	38.4	18.1	18.1	63.9	−0.202	0.000	792
ND5	29.0	39.9	14.7	16.5	68.9	−0.158	−0.058	1720
ND4	28.3	40.3	16.6	14.7	68.6	−0.175	0.060	1431
ND4L	24.8	43.9	16.2	15.2	68.6	−0.279	0.032	303
ND6	27.9	44.1	14.3	13.7	72.1	−0.225	0.021	519
Cyt b	25.6	39.3	17.2	17.9	64.9	−0.211	−0.018	1137
ND1	23.9	42.5	18.8	14.8	66.3	−0.280	0.118	930
ND3	27.3	41.4	14.9	16.4	68.7	−0.205	−0.046	348
tRNAs	35.2	34.2	17.4	13.3	69.3	0.015	0.135	1455
rRNAs	37.1	36.2	15.8	10.9	73.3	0.011	0.185	2205
CR	32.5	29.9	19.1	18.5	62.4	0.042	0.014	1495

Table S2. Composition and skewness of *D. aspersus* mitogenome.

	A%	T%	G%	C%	(A + T) %	AT-skew	GC-skew	Length(bp)
Mitogenome	33.4	32.6	15.7	18.3	66.0	0.011	−0.076	16,916
PCGs	26.2	38.7	17.2	17.9	64.9	−0.193	−0.020	11,224
COI	26.3	36.4	18.6	18.7	62.7	−0.161	−0.003	1539
COII	29.5	35.0	18.5	17.1	64.5	−0.085	0.041	693
ND2	26.6	39.3	15.1	19.0	66.0	−0.192	−0.114	1002
ATP8	39.6	35.2	8.2	17.0	74.8	0.059	−0.350	159
ATP6	26.7	37.5	14.1	21.8	64.1	−0.169	−0.215	675
COIII	24.1	37.1	18.6	20.2	61.2	−0.212	−0.042	792
ND6	29.3	39.1	13.7	17.9	68.4	−0.144	−0.134	519
Cyt b	26.3	37.1	16.8	19.8	63.4	−0.169	−0.082	1137
ND5	26.5	41.2	16.7	15.6	67.7	−0.216	0.034	1726
ND4	24.1	40.3	19.7	15.9	64.5	−0.251	0.106	1404
ND4L	22.4	41.9	18.8	16.8	64.4	−0.303	0.056	303
ND1	24.3	40.1	18.6	17.0	64.4	−0.245	0.045	930
ND3	24.3	41.7	15.9	18.0	66.1	−0.263	−0.060	345
tRNAs	34.8	34.0	17.8	13.4	68.8	0.012	0.140	1460
rRNAs	33.7	36.5	18.6	11.3	70.1	−0.040	0.245	2201
CR	30.9	33.0	17.7	18.4	63.9	−0.033	−0.019	1461

Table S3. Relative synonymous codon usage in the mitogenomes of 15 Paguroidea species.

Species name	Codon	RSCU	Codon	RSCU	Codon	RSCU	Codon	RSCU
Pagurus filholi	UUU(F)	1.65	UCU(S)	3.13	UAU(Y)	1.72	UGU(C)	1.90
	UUC(F)	0.35	UCC(S)	0.31	UAC(Y)	0.28	UGC(C)	0.10
	UUA(L)	3.82	UCA(S)	1.65	UAA(*)	0.00	UGA(W)	1.80
	UUG(L)	0.51	UCG(S)	0.10	UAG(*)	0.00	UGG(W)	0.20
	CUU(L)	0.93	CCU(P)	2.04	CAU(H)	1.42	CGU(R)	0.92
	CUC(L)	0.17	CCC(P)	0.46	CAC(H)	0.58	CGC(R)	0.26
	CUA(L)	0.51	CCA(P)	1.41	CAA(Q)	1.79	CGA(R)	2.43
	CUG(L)	0.06	CCG(P)	0.08	CAG(Q)	0.21	CGG(R)	0.39
	AUU(I)	1.77	ACU(T)	1.80	AAU(N)	1.65	AGU(S)	0.85
	AUC(I)	0.23	ACC(T)	0.50	AAC(N)	0.35	AGC(S)	0.15
	AUA(M)	1.70	ACA(T)	1.59	AAA(K)	1.86	AGA(S)	1.44
	AUG(M)	0.30	ACG(T)	0.11	AAG(K)	0.14	AGG(S)	0.38
	GUU(V)	1.75	GCU(A)	1.78	GAU(D)	1.65	GGU(G)	1.32
	GUC(V)	0.20	GCC(A)	0.71	GAC(D)	0.35	GGC(G)	0.26
	GUA(V)	1.85	GCA(A)	1.26	GAA(E)	1.75	GGA(G)	1.96
	GUG(V)	0.20	GCG(A)	0.24	GAG(E)	0.25	GGG(G)	0.46
Pagurus japonicus	UUU(F)	1.66	UCU(S)	3.11	UAU(Y)	1.74	UGU(C)	1.91
	UUC(F)	0.34	UCC(S)	0.29	UAC(Y)	0.26	UGC(C)	0.09
	UUA(L)	3.86	UCA(S)	1.68	UAA(*)	0.00	UGA(W)	1.82
	UUG(L)	0.45	UCG(S)	0.10	UAG(*)	0.00	UGG(W)	0.18
	CUU(L)	0.93	CCU(P)	2.01	CAU(H)	1.47	CGU(R)	0.92
	CUC(L)	0.18	CCC(P)	0.49	CAC(H)	0.53	CGC(R)	0.26
	CUA(L)	0.52	CCA(P)	1.39	CAA(Q)	1.79	CGA(R)	2.43
	CUG(L)	0.06	CCG(P)	0.11	CAG(Q)	0.21	CGG(R)	0.39
	AUU(I)	1.76	ACU(T)	1.80	AAU(N)	1.64	AGU(S)	0.84
	AUC(I)	0.24	ACC(T)	0.49	AAC(N)	0.36	AGC(S)	0.15
	AUA(M)	1.70	ACA(T)	1.60	AAA(K)	1.84	AGA(S)	1.45
	AUG(M)	0.30	ACG(T)	0.11	AAG(K)	0.16	AGG(S)	0.38
	GUU(V)	1.76	GCU(A)	1.86	GAU(D)	1.68	GGU(G)	1.27
	GUC(V)	0.20	GCC(A)	0.63	GAC(D)	0.32	GGC(G)	0.27
	GUA(V)	1.86	GCA(A)	1.28	GAA(E)	1.80	GGA(G)	2.00
	GUG(V)	0.18	GCG(A)	0.23	GAG(E)	0.20	GGG(G)	0.46
Pagurus gracilipes	UUU(F)	1.72	UCU(S)	2.81	UAU(Y)	1.77	UGU(C)	1.92
	UUC(F)	0.28	UCC(S)	0.50	UAC(Y)	0.23	UGC(C)	0.08
	UUA(L)	3.82	UCA(S)	1.78	UAA(*)	0.00	UGA(W)	1.74
	UUG(L)	0.49	UCG(S)	0.08	UAG(*)	0.00	UGG(W)	0.26
	CUU(L)	0.91	CCU(P)	1.94	CAU(H)	1.46	CGU(R)	1.03
	CUC(L)	0.14	CCC(P)	0.51	CAC(H)	0.54	CGC(R)	0.28
	CUA(L)	0.55	CCA(P)	1.31	CAA(Q)	1.65	CGA(R)	2.00
	CUG(L)	0.09	CCG(P)	0.23	CAG(Q)	0.35	CGG(R)	0.69

	AUU(I)	1.76	ACU(T)	1.82	AAU(N)	1.71	AGU(S)	0.77
	AUC(I)	0.24	ACC(T)	0.53	AAC(N)	0.29	AGC(S)	0.15
	AUA(M)	1.69	ACA(T)	1.54	AAA(K)	1.76	AGA(S)	1.65
	AUG(M)	0.31	ACG(T)	0.11	AAG(K)	0.24	AGG(S)	0.25
	GUU(V)	1.84	GCU(A)	1.82	GAU(D)	1.61	GGU(G)	1.39
	GUC(V)	0.17	GCC(A)	0.59	GAC(D)	0.39	GGC(G)	0.18
	GUA(V)	1.77	GCA(A)	1.32	GAA(E)	1.58	GGA(G)	1.86
	GUG(V)	0.22	GCG(A)	0.27	GAG(E)	0.42	GGG(G)	0.57
Pagurus maculosus	UUU(F)	1.76	UCU(S)	3.09	UAU(Y)	1.75	UGU(C)	1.95
	UUC(F)	0.24	UCC(S)	0.18	UAC(Y)	0.25	UGC(C)	0.05
	UUA(L)	4.00	UCA(S)	1.80	UAA(*)	0.00	UGA(W)	1.80
	UUG(L)	0.42	UCG(S)	0.11	UAG(*)	0.00	UGG(W)	0.20
	CUU(L)	0.96	CCU(P)	2.10	CAU(H)	1.61	CGU(R)	1.29
	CUC(L)	0.04	CCC(P)	0.37	CAC(H)	0.39	CGC(R)	0.14
	CUA(L)	0.53	CCA(P)	1.38	CAA(Q)	1.71	CGA(R)	2.24
	CUG(L)	0.04	CCG(P)	0.14	CAG(Q)	0.29	CGG(R)	0.34
	AUU(I)	1.90	ACU(T)	2.11	AAU(N)	1.82	AGU(S)	0.84
	AUC(I)	0.10	ACC(T)	0.36	AAC(N)	0.18	AGC(S)	0.11
	AUA(M)	1.72	ACA(T)	1.51	AAA(K)	1.86	AGA(S)	1.60
	AUG(M)	0.28	ACG(T)	0.02	AAG(K)	0.14	AGG(S)	0.27
	GUU(V)	1.85	GCU(A)	2.02	GAU(D)	1.84	GGU(G)	1.27
	GUC(V)	0.09	GCC(A)	0.46	GAC(D)	0.16	GGC(G)	0.07
	GUA(V)	1.82	GCA(A)	1.39	GAA(E)	1.69	GGA(G)	2.40
	GUG(V)	0.24	GCG(A)	0.14	GAG(E)	0.31	GGG(G)	0.26
Pagurus nigrofascia	UUU(F)	1.76	UCU(S)	2.96	UAU(Y)	1.69	UGU(C)	1.71
	UUC(F)	0.24	UCC(S)	0.30	UAC(Y)	0.31	UGC(C)	0.29
	UUA(L)	3.19	UCA(S)	1.63	UAA(*)	0.00	UGA(W)	1.74
	UUG(L)	1.01	UCG(S)	0.32	UAG(*)	0.00	UGG(W)	0.26
	CUU(L)	0.94	CCU(P)	1.67	CAU(H)	1.36	CGU(R)	0.73
	CUC(L)	0.16	CCC(P)	0.81	CAC(H)	0.64	CGC(R)	0.40
	CUA(L)	0.51	CCA(P)	1.29	CAA(Q)	1.40	CGA(R)	2.20
	CUG(L)	0.19	CCG(P)	0.23	CAG(Q)	0.60	CGG(R)	0.67
	AUU(I)	1.73	ACU(T)	1.71	AAU(N)	1.53	AGU(S)	0.73
	AUC(I)	0.27	ACC(T)	0.47	AAC(N)	0.47	AGC(S)	0.13
	AUA(M)	1.45	ACA(T)	1.66	AAA(K)	1.60	AGA(S)	1.46
	AUG(M)	0.55	ACG(T)	0.16	AAG(K)	0.40	AGG(S)	0.47
	GUU(V)	1.75	GCU(A)	1.63	GAU(D)	1.51	GGU(G)	1.23
	GUC(V)	0.27	GCC(A)	0.73	GAC(D)	0.49	GGC(G)	0.28
	GUA(V)	1.47	GCA(A)	1.35	GAA(E)	1.36	GGA(G)	2.06
	GUG(V)	0.52	GCG(A)	0.28	GAG(E)	0.64	GGG(G)	0.44
Pagurus longicarpus	UUU(F)	1.52	UCU(S)	3.09	UAU(Y)	1.71	UGU(C)	1.85
	UUC(F)	0.48	UCC(S)	0.51	UAC(Y)	0.29	UGC(C)	0.15

	UUA(L)	3.08	UCA(S)	1.55	UAA(*)	0.00	UGA(W)	1.86
	UUG(L)	0.50	UCG(S)	0.19	UAG(*)	0.00	UGG(W)	0.14
	CUU(L)	1.06	CCU(P)	1.73	CAU(H)	1.29	CGU(R)	1.53
	CUC(L)	0.18	CCC(P)	0.48	CAC(H)	0.71	CGC(R)	0.00
	CUA(L)	1.04	CCA(P)	1.73	CAA(Q)	1.69	CGA(R)	2.13
	CUG(L)	0.15	CCG(P)	0.06	CAG(Q)	0.31	CGG(R)	0.33
	AUU(I)	1.81	ACU(T)	1.81	AAU(N)	1.51	AGU(S)	0.70
	AUC(I)	0.19	ACC(T)	0.36	AAC(N)	0.49	AGC(S)	0.00
	AUA(M)	1.57	ACA(T)	1.77	AAA(K)	1.72	AGA(S)	1.60
	AUG(M)	0.43	ACG(T)	0.07	AAG(K)	0.28	AGG(S)	0.36
	GUU(V)	1.80	GCU(A)	1.83	GAU(D)	1.48	GGU(G)	1.70
	GUC(V)	0.11	GCC(A)	0.54	GAC(D)	0.52	GGC(G)	0.12
	GUA(V)	1.80	GCA(A)	1.45	GAA(E)	1.29	GGA(G)	1.71
	GUG(V)	0.28	GCG(A)	0.18	GAG(E)	0.71	GGG(G)	0.47
Coenobita rugosus	UUU(F)	1.44	UCU(S)	2.50	UAU(Y)	1.13	UGU(C)	1.24
	UUC(F)	0.56	UCC(S)	0.78	UAC(Y)	0.87	UGC(C)	0.76
	UUA(L)	1.62	UCA(S)	1.10	UAA(*)	0.00	UGA(W)	1.01
	UUG(L)	1.29	UCG(S)	0.57	UAG(*)	0.00	UGG(W)	0.99
	CUU(L)	0.88	CCU(P)	1.70	CAU(H)	1.41	CGU(R)	0.85
	CUC(L)	0.62	CCC(P)	1.12	CAC(H)	0.59	CGC(R)	0.59
	CUA(L)	1.19	CCA(P)	0.66	CAA(Q)	0.94	CGA(R)	1.57
	CUG(L)	0.40	CCG(P)	0.52	CAG(Q)	1.06	CGG(R)	0.98
	AUU(I)	1.52	ACU(T)	1.71	AAU(N)	0.98	AGU(S)	0.69
	AUC(I)	0.48	ACC(T)	0.92	AAC(N)	1.02	AGC(S)	0.32
	AUA(M)	0.99	ACA(T)	0.98	AAA(K)	1.32	AGA(S)	1.31
	AUG(M)	1.01	ACG(T)	0.39	AAG(K)	0.68	AGG(S)	0.73
	GUU(V)	1.79	GCU(A)	1.53	GAU(D)	1.11	GGU(G)	0.81
	GUC(V)	0.62	GCC(A)	1.14	GAC(D)	0.89	GGC(G)	0.41
	GUA(V)	0.82	GCA(A)	0.84	GAA(E)	1.08	GGA(G)	1.39
	GUG(V)	0.78	GCG(A)	0.49	GAG(E)	0.92	GGG(G)	1.39
Coenobita variabilis	UUU(F)	1.60	UCU(S)	2.36	UAU(Y)	1.34	UGU(C)	1.30
	UUC(F)	0.40	UCC(S)	0.85	UAC(Y)	0.66	UGC(C)	0.70
	UUA(L)	1.65	UCA(S)	1.28	UAA(*)	0.00	UGA(W)	1.09
	UUG(L)	1.24	UCG(S)	0.47	UAG(*)	0.00	UGG(W)	0.91
	CUU(L)	0.90	CCU(P)	1.95	CAU(H)	1.65	CGU(R)	1.05
	CUC(L)	0.54	CCC(P)	0.82	CAC(H)	0.35	CGC(R)	0.26
	CUA(L)	1.41	CCA(P)	0.85	CAA(Q)	1.04	CGA(R)	1.90
	CUG(L)	0.27	CCG(P)	0.38	CAG(Q)	0.96	CGG(R)	0.79
	AUU(I)	1.51	ACU(T)	1.95	AAU(N)	1.28	AGU(S)	0.52
	AUC(I)	0.49	ACC(T)	0.74	AAC(N)	0.72	AGC(S)	0.36
	AUA(M)	1.18	ACA(T)	0.91	AAA(K)	1.33	AGA(S)	1.55
	AUG(M)	0.82	ACG(T)	0.41	AAG(K)	0.67	AGG(S)	0.61

	GUU(V)	1.83	GCU(A)	1.51	GAU(D)	1.42	GGU(G)	0.82
	GUC(V)	0.40	GCC(A)	1.00	GAC(D)	0.58	GGC(G)	0.44
	GUA(V)	0.89	GCA(A)	1.06	GAA(E)	1.27	GGA(G)	1.74
	GUG(V)	0.88	GCG(A)	0.43	GAG(E)	0.73	GGG(G)	1.00
Coenobita perlatus	UUU(F)	1.57	UCU(S)	2.52	UAU(Y)	1.31	UGU(C)	1.49
	UUC(F)	0.43	UCC(S)	0.67	UAC(Y)	0.69	UGC(C)	0.51
	UUA(L)	2.28	UCA(S)	1.51	UAA(*)	0.00	UGA(W)	1.32
	UUG(L)	1.02	UCG(S)	0.34	UAG(*)	0.00	UGG(W)	0.68
	CUU(L)	1.04	CCU(P)	2.72	CAU(H)	1.64	CGU(R)	1.11
	CUC(L)	0.17	CCC(P)	0.20	CAC(H)	0.36	CGC(R)	0.07
	CUA(L)	1.34	CCA(P)	0.91	CAA(Q)	1.34	CGA(R)	1.90
	CUG(L)	0.15	CCG(P)	0.17	CAG(Q)	0.66	CGG(R)	0.92
	AUU(I)	1.69	ACU(T)	2.02	AAU(N)	1.38	AGU(S)	0.47
	AUC(I)	0.31	ACC(T)	0.55	AAC(N)	0.62	AGC(S)	0.13
	AUA(M)	1.44	ACA(T)	1.19	AAA(K)	1.26	AGA(S)	1.75
	AUG(M)	0.56	ACG(T)	0.24	AAG(K)	0.74	AGG(S)	0.61
	GUU(V)	1.85	GCU(A)	2.25	GAU(D)	1.55	GGU(G)	1.25
	GUC(V)	0.33	GCC(A)	0.48	GAC(D)	0.45	GGC(G)	0.24
	GUA(V)	1.30	GCA(A)	1.00	GAA(E)	1.33	GGA(G)	1.67
	GUG(V)	0.52	GCG(A)	0.26	GAG(E)	0.67	GGG(G)	0.84
Coenobita brevimanus	UUU(F)	1.43	UCU(S)	2.26	UAU(Y)	1.35	UGU(C)	1.20
	UUC(F)	0.57	UCC(S)	0.85	UAC(Y)	0.65	UGC(C)	0.80
	UUA(L)	1.80	UCA(S)	1.17	UAA(*)	0.00	UGA(W)	1.20
	UUG(L)	1.30	UCG(S)	0.61	UAG(*)	0.00	UGG(W)	0.80
	CUU(L)	0.94	CCU(P)	2.28	CAU(H)	1.73	CGU(R)	1.11
	CUC(L)	0.42	CCC(P)	0.44	CAC(H)	0.27	CGC(R)	0.20
	CUA(L)	1.32	CCA(P)	0.75	CAA(Q)	1.09	CGA(R)	1.44
	CUG(L)	0.23	CCG(P)	0.53	CAG(Q)	0.91	CGG(R)	1.25
	AUU(I)	1.56	ACU(T)	2.15	AAU(N)	1.31	AGU(S)	0.43
	AUC(I)	0.44	ACC(T)	0.60	AAC(N)	0.69	AGC(S)	0.22
	AUA(M)	1.07	ACA(T)	0.78	AAA(K)	1.31	AGA(S)	1.68
	AUG(M)	0.93	ACG(T)	0.47	AAG(K)	0.69	AGG(S)	0.78
	GUU(V)	1.77	GCU(A)	2.00	GAU(D)	1.26	GGU(G)	1.02
	GUC(V)	0.41	GCC(A)	0.67	GAC(D)	0.74	GGC(G)	0.35
	GUA(V)	1.08	GCA(A)	0.91	GAA(E)	1.31	GGA(G)	1.47
	GUG(V)	0.74	GCG(A)	0.43	GAG(E)	0.69	GGG(G)	1.16
Birgus latro	UUU(F)	1.44	UCU(S)	2.48	UAU(Y)	1.29	UGU(C)	1.63
	UUC(F)	0.56	UCC(S)	0.86	UAC(Y)	0.71	UGC(C)	0.37
	UUA(L)	2.15	UCA(S)	1.31	UAA(*)	0.00	UGA(W)	1.40
	UUG(L)	0.92	UCG(S)	0.29	UAG(*)	0.00	UGG(W)	0.60
	CUU(L)	0.97	CCU(P)	1.91	CAU(H)	1.10	CGU(R)	1.11
	CUC(L)	0.33	CCC(P)	0.62	CAC(H)	0.90	CGC(R)	0.39

	CUA(L)	1.41	CCA(P)	1.26	CAA(Q)	1.43	CGA(R)	2.10
	CUG(L)	0.22	CCG(P)	0.21	CAG(Q)	0.57	CGG(R)	0.39
	AUU(I)	1.49	ACU(T)	1.80	AAU(N)	1.19	AGU(S)	0.65
	AUC(I)	0.51	ACC(T)	0.68	AAC(N)	0.81	AGC(S)	0.20
	AUA(M)	1.33	ACA(T)	1.39	AAA(K)	1.39	AGA(S)	1.62
	AUG(M)	0.67	ACG(T)	0.14	AAG(K)	0.61	AGG(S)	0.59
	GUU(V)	1.65	GCU(A)	2.00	GAU(D)	1.27	GGU(G)	1.15
	GUC(V)	0.48	GCC(A)	0.82	GAC(D)	0.73	GGC(G)	0.29
	GUA(V)	1.46	GCA(A)	1.04	GAA(E)	1.30	GGA(G)	1.66
	GUG(V)	0.42	GCG(A)	0.14	GAG(E)	0.70	GGG(G)	0.91
Dardanus arrosor	UUU(F)	1.74	UCU(S)	3.15	UAU(Y)	1.52	UGU(C)	1.42
	UUC(F)	0.26	UCC(S)	0.24	UAC(Y)	0.48	UGC(C)	0.58
	UUA(L)	2.77	UCA(S)	1.06	UAA(*)	0.00	UGA(W)	1.52
	UUG(L)	0.40	UCG(S)	0.21	UAG(*)	0.00	UGG(W)	0.48
	CUU(L)	1.63	CCU(P)	1.82	CAU(H)	1.28	CGU(R)	0.80
	CUC(L)	0.39	CCC(P)	0.77	CAC(H)	0.72	CGC(R)	0.40
	CUA(L)	0.65	CCA(P)	0.99	CAA(Q)	1.53	CGA(R)	2.00
	CUG(L)	0.17	CCG(P)	0.41	CAG(Q)	0.47	CGG(R)	0.80
	AUU(I)	1.80	ACU(T)	1.81	AAU(N)	1.66	AGU(S)	0.59
	AUC(I)	0.20	ACC(T)	0.46	AAC(N)	0.34	AGC(S)	0.38
	AUA(M)	1.45	ACA(T)	1.60	AAA(K)	1.71	AGA(S)	1.86
	AUG(M)	0.55	ACG(T)	0.13	AAG(K)	0.29	AGG(S)	0.52
	GUU(V)	2.15	GCU(A)	1.58	GAU(D)	1.61	GGU(G)	0.66
	GUC(V)	0.50	GCC(A)	0.56	GAC(D)	0.39	GGC(G)	0.65
	GUA(V)	0.92	GCA(A)	1.41	GAA(E)	1.39	GGA(G)	1.45
	GUG(V)	0.44	GCG(A)	0.46	GAG(E)	0.61	GGG(G)	1.24
Dardanus aspersus	UUU(F)	1.53	UCU(S)	2.67	UAU(Y)	1.55	UGU(C)	1.61
	UUC(F)	0.47	UCC(S)	0.86	UAC(Y)	0.45	UGC(C)	0.39
	UUA(L)	2.25	UCA(S)	1.01	UAA(*)	0.00	UGA(W)	1.41
	UUG(L)	0.61	UCG(S)	0.22	UAG(*)	0.00	UGG(W)	0.59
	CUU(L)	1.51	CCU(P)	1.73	CAU(H)	1.26	CGU(R)	0.85
	CUC(L)	0.58	CCC(P)	1.15	CAC(H)	0.74	CGC(R)	0.46
	CUA(L)	0.76	CCA(P)	0.73	CAA(Q)	1.48	CGA(R)	1.97
	CUG(L)	0.29	CCG(P)	0.39	CAG(Q)	0.52	CGG(R)	0.72
	AUU(I)	1.54	ACU(T)	1.49	AAU(N)	1.47	AGU(S)	0.67
	AUC(I)	0.46	ACC(T)	0.79	AAC(N)	0.53	AGC(S)	0.26
	AUA(M)	1.62	ACA(T)	1.43	AAA(K)	1.58	AGA(S)	1.54
	AUG(M)	0.38	ACG(T)	0.30	AAG(K)	0.42	AGG(S)	0.77
	GUU(V)	1.94	GCU(A)	1.58	GAU(D)	1.31	GGU(G)	0.90
	GUC(V)	0.51	GCC(A)	0.77	GAC(D)	0.69	GGC(G)	0.61
	GUA(V)	1.07	GCA(A)	1.23	GAA(E)	1.23	GGA(G)	1.34
	GUG(V)	0.48	GCG(A)	0.42	GAG(E)	0.77	GGG(G)	1.15

Clibanarius infraspinatus	UUU(F)	1.66	UCU(S)	2.75	UAU(Y)	1.61	UGU(C)	1.77
	UUC(F)	0.34	UCC(S)	0.43	UAC(Y)	0.39	UGC(C)	0.23
	UUA(L)	2.58	UCA(S)	1.42	UAA(*)	0.00	UGA(W)	1.27
	UUG(L)	1.17	UCG(S)	0.18	UAG(*)	0.00	UGG(W)	0.73
	CUU(L)	0.92	CCU(P)	1.49	CAU(H)	0.88	CGU(R)	0.61
	CUC(L)	0.30	CCC(P)	0.88	CAC(H)	1.12	CGC(R)	0.27
	CUA(L)	0.83	CCA(P)	1.60	CAA(Q)	1.66	CGA(R)	2.64
	CUG(L)	0.19	CCG(P)	0.03	CAG(Q)	0.34	CGG(R)	0.47
	AUU(I)	1.59	ACU(T)	1.43	AAU(N)	1.29	AGU(S)	0.79
	AUC(I)	0.41	ACC(T)	0.74	AAC(N)	0.71	AGC(S)	0.25
	AUA(M)	1.46	ACA(T)	1.65	AAA(K)	1.59	AGA(S)	1.35
	AUG(M)	0.54	ACG(T)	0.18	AAG(K)	0.41	AGG(S)	0.83
	GUU(V)	1.89	GCU(A)	1.75	GAU(D)	1.38	GGU(G)	1.43
	GUC(V)	0.30	GCC(A)	0.75	GAC(D)	0.62	GGC(G)	0.34
	GUA(V)	1.22	GCA(A)	1.30	GAA(E)	1.21	GGA(G)	1.20
	GUG(V)	0.58	GCG(A)	0.21	GAG(E)	0.79	GGG(G)	1.02
Pylocheles mortensenii	UUU(F)	1.76	UCU(S)	2.61	UAU(Y)	1.95	UGU(C)	2.00
	UUC(F)	0.24	UCC(S)	0.36	UAC(Y)	0.05	UGC(C)	0.00
	UUA(L)	4.51	UCA(S)	1.94	UAA(*)	0.00	UGA(W)	1.87
	UUG(L)	0.19	UCG(S)	0.04	UAG(*)	0.00	UGG(W)	0.13
	CUU(L)	0.88	CCU(P)	2.71	CAU(H)	1.83	CGU(R)	1.10
	CUC(L)	0.07	CCC(P)	0.08	CAC(H)	0.17	CGC(R)	0.14
	CUA(L)	0.32	CCA(P)	1.15	CAA(Q)	1.85	CGA(R)	2.55
	CUG(L)	0.03	CCG(P)	0.06	CAG(Q)	0.15	CGG(R)	0.21
	AUU(I)	1.89	ACU(T)	1.99	AAU(N)	1.81	AGU(S)	0.94
	AUC(I)	0.11	ACC(T)	0.25	AAC(N)	0.19	AGC(S)	0.07
	AUA(M)	1.91	ACA(T)	1.76	AAA(K)	1.81	AGA(S)	1.74
	AUG(M)	0.09	ACG(T)	0.00	AAG(K)	0.19	AGG(S)	0.31
	GUU(V)	1.89	GCU(A)	2.49	GAU(D)	1.83	GGU(G)	1.32
	GUC(V)	0.02	GCC(A)	0.22	GAC(D)	0.17	GGC(G)	0.04
	GUA(V)	1.93	GCA(A)	1.24	GAA(E)	1.67	GGA(G)	2.40
	GUG(V)	0.16	GCG(A)	0.04	GAG(E)	0.33	GGG(G)	0.25

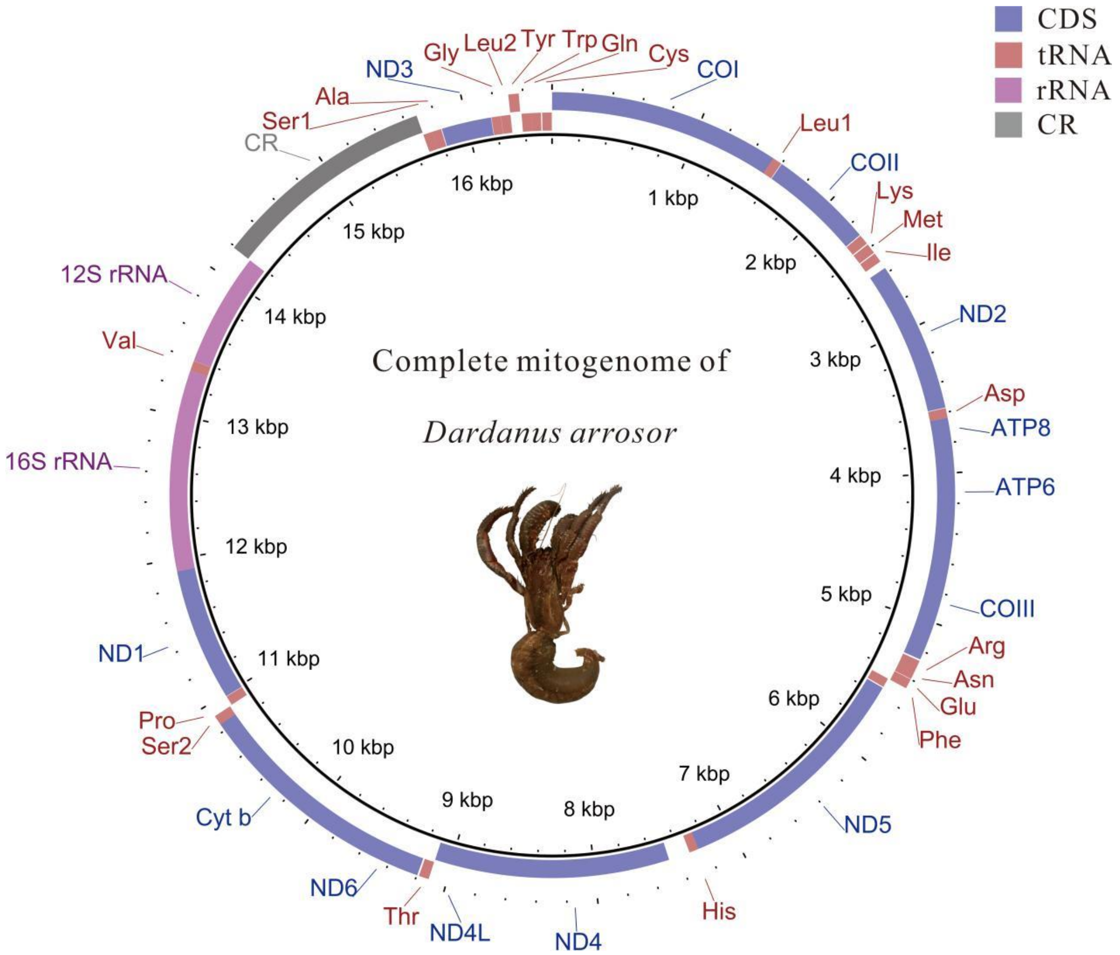

Fig S1. Gene map of the *D. arrosor* mitogenome. Genes outside the circular indicating they are encoded by the heavy strand; genes inside the circular indicating they are encoded by the light strand.

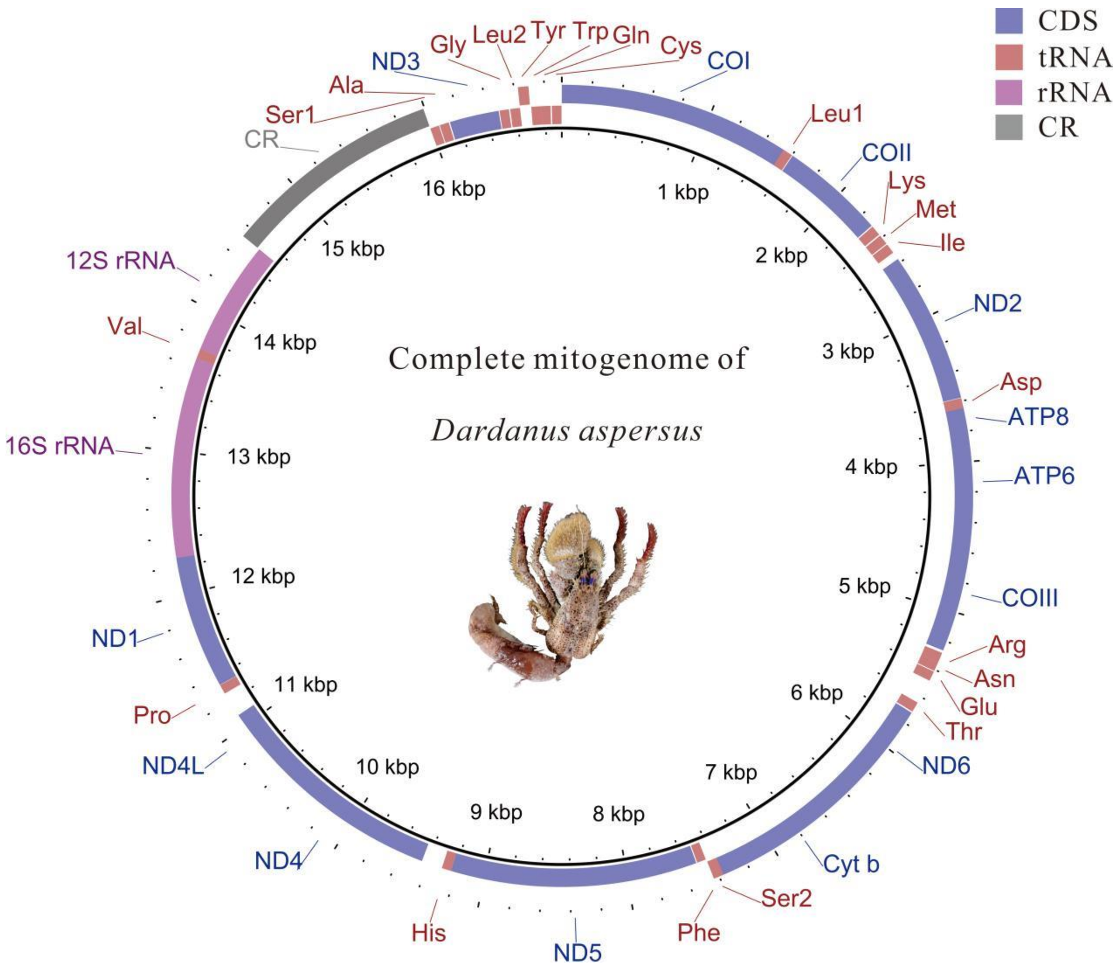

Fig S2. Gene map of the *D. aspersus* mitogenome. Genes outside the circular indicating they are encoded by the heavy strand; genes inside the circular indicating they are encoded by the light strand.

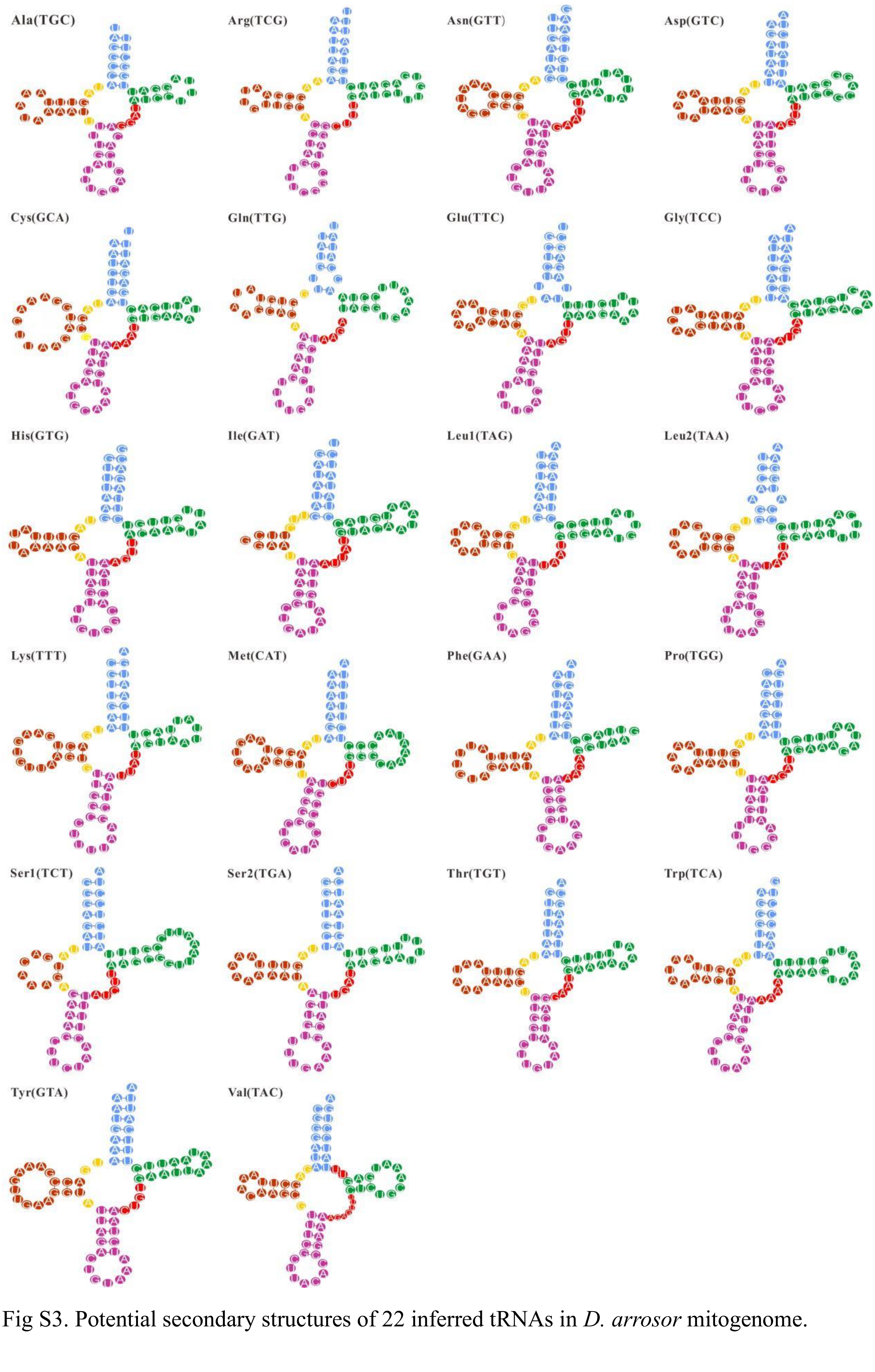

Fig S3. Potential secondary structures of 22 inferred tRNAs in *D. arrosor* mitogenome.

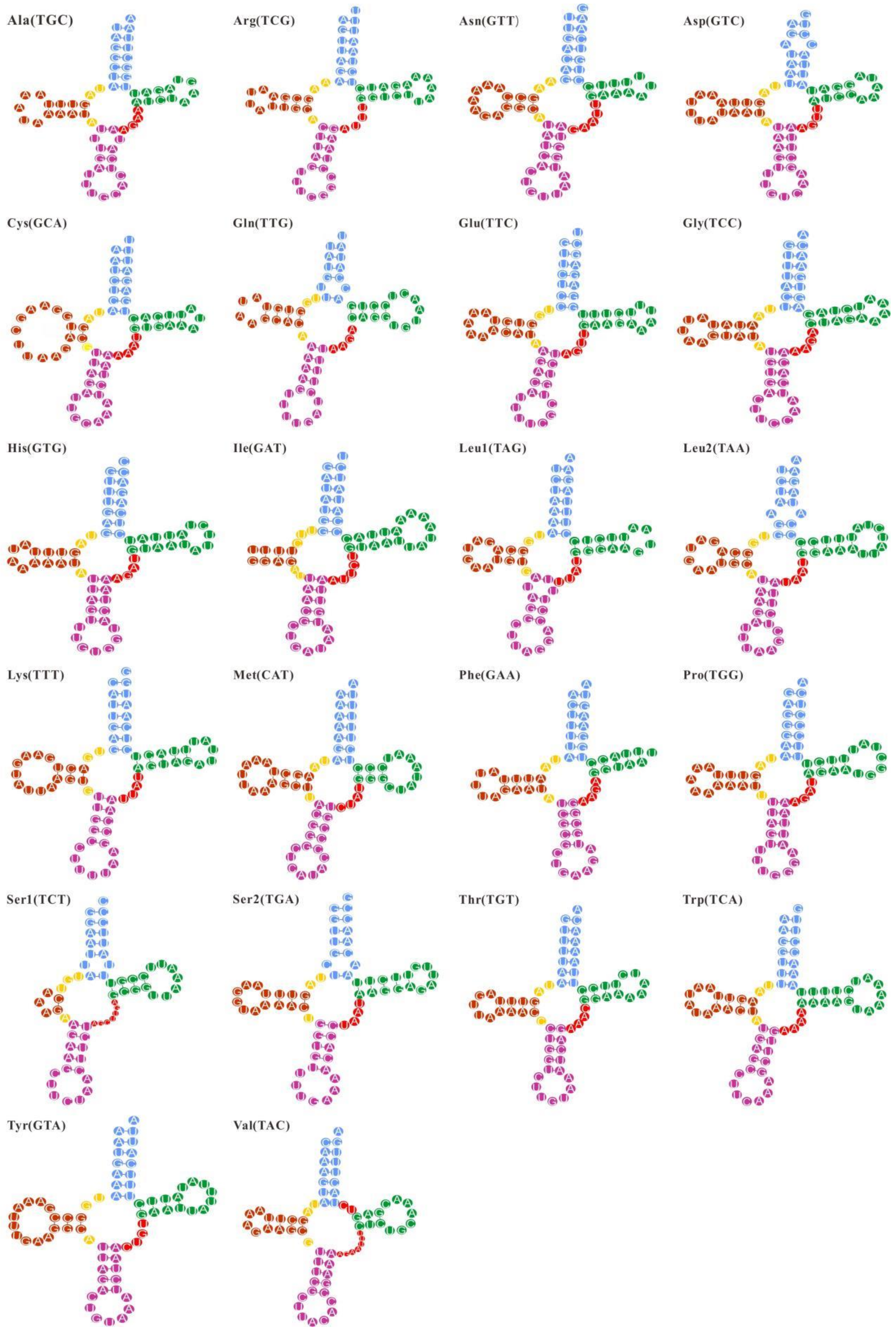

Fig S4. Potential secondary structures of 22 inferred tRNAs in *D. aspersus* mitogenome.

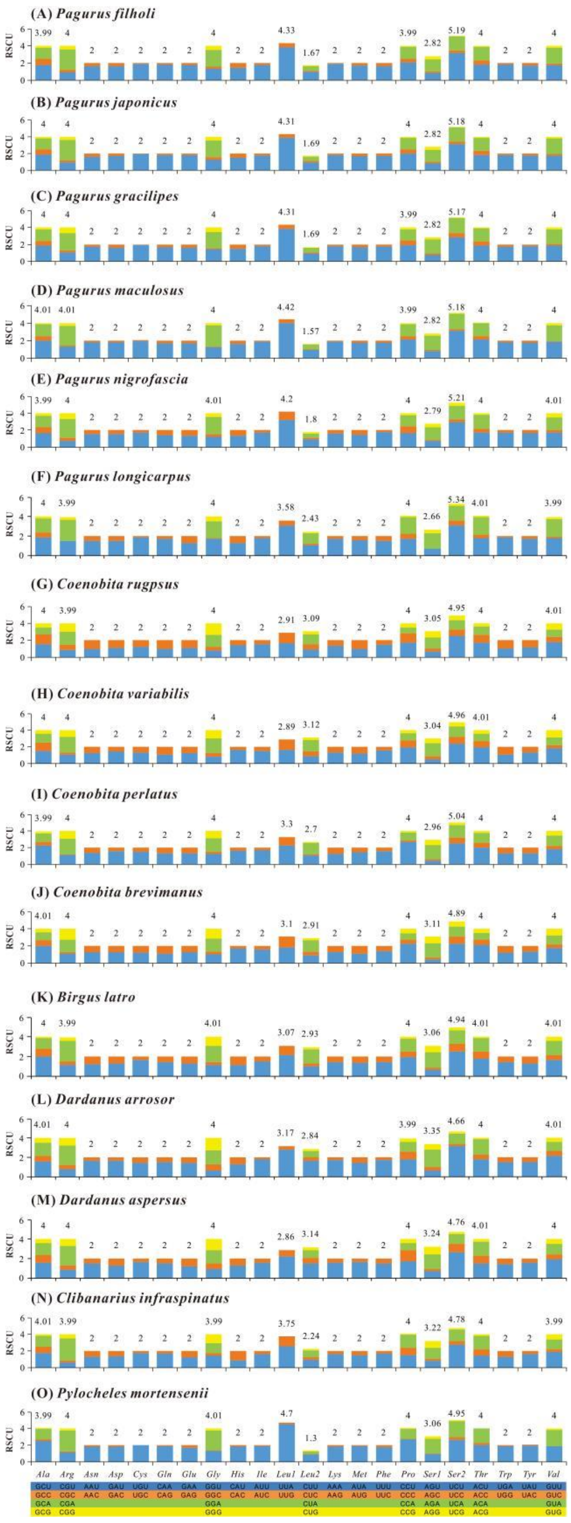

Fig S5. Relative synonymous codon usage (RSCU) in the mitogenomes of 15 Paguroidea species.

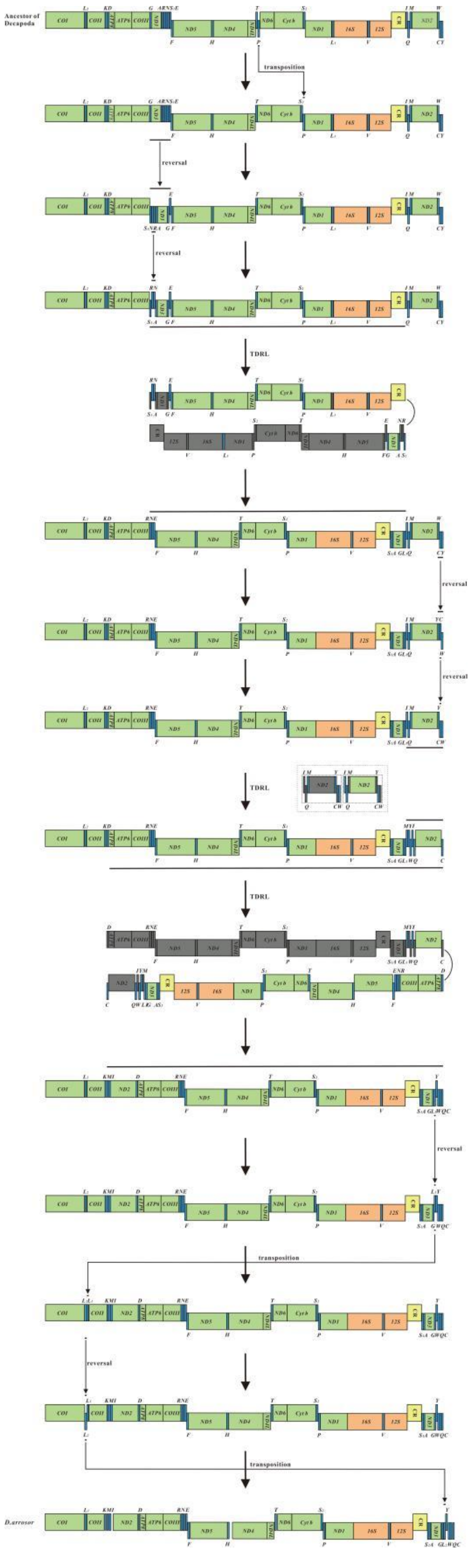

Fig S6. Inferred intermediate steps between the ancestral gene arrangement of crustaceans and *D. arrosor* mitogenome.

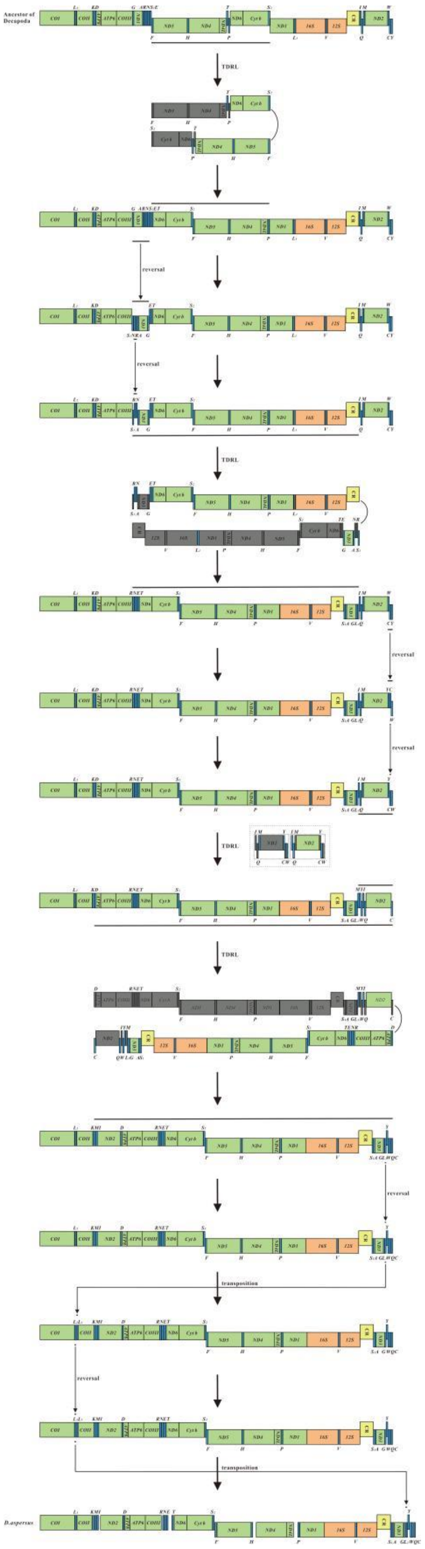

Fig S7. Inferred intermediate steps between the ancestral gene arrangement of crustaceans and *D. aspersus* mitogenome.
